# Supplementary figures and images for: Constitutive expression of full-length or partial of SOC1 genes for yield enhancement in tomato
Source: Front Plant Sci. 2025 Jul 28;16:1640731. doi: 10.3389/fpls.2025.1640731 (PMC12336250; doi:10.3389/fpls.2025.1640731)

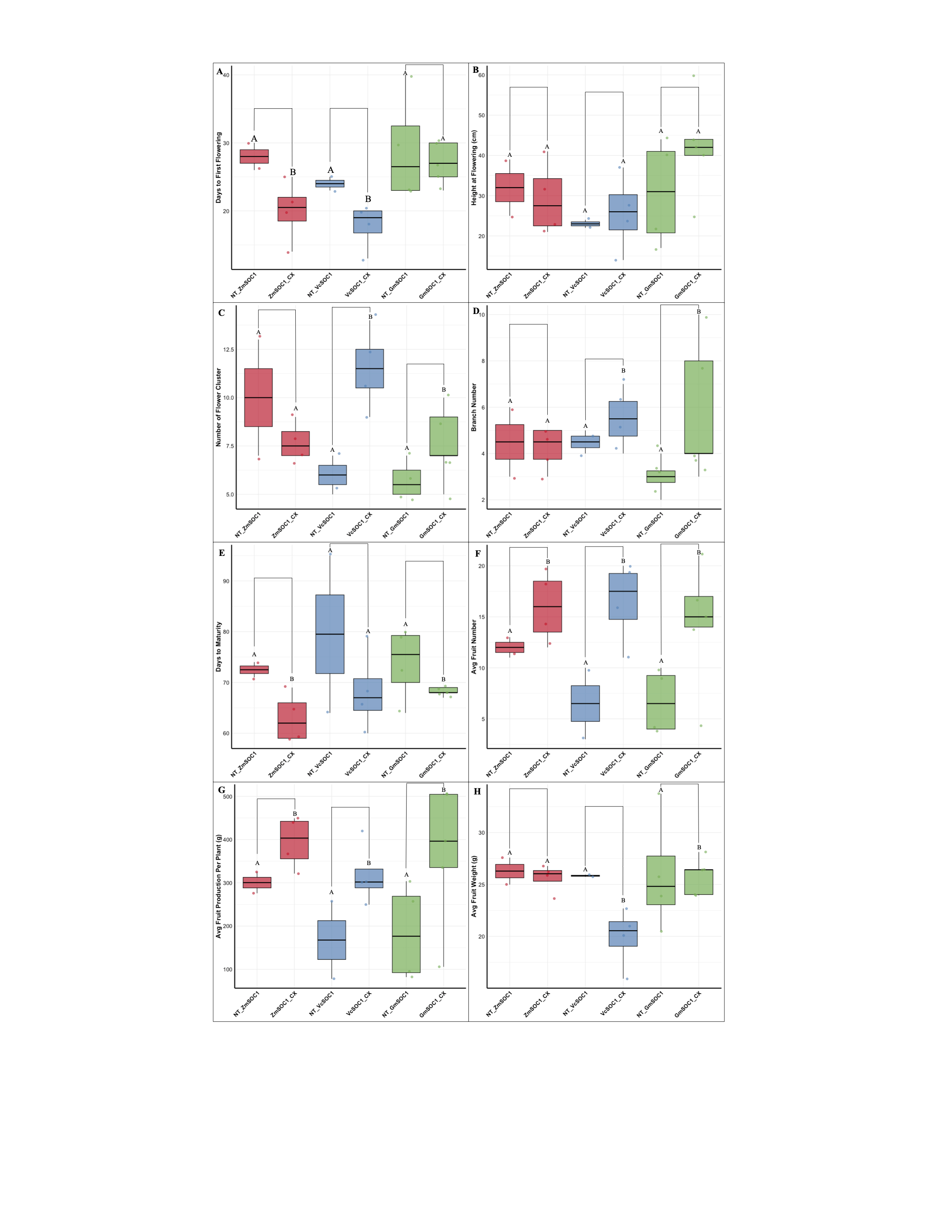

Supplement: Supplementary Figure 1 — Phenotypic comparisons of T0 transgenic lines (GmSOC1_CX, n = 5; ZmSOC1_CX, n = 4; and VcSOC1K_CX, n = 3) and their corresponding non-transgenic (NT) lines (NT_GmSOC1, n = 4; NT_ZmSOC1, n = 3; and NT_VcSOC1K, n = 3). (A) Days to the appearance of the first flower after potting in a one-gallon pot. (B) Plant height at the time of first flowering. (C) Number of flower clusters counted after all fruits were harvested. (D) Number of branches counted after all fruits were harvested. (E) Days to the appearance of the first mature fruit after potting in a one-gallon pot. (F) Total number of fruits harvested. (G) Total weight of harvested fruits. (H) Average weight per fruit. The y-axis shows averages, and bars indicate standard deviations. [file Image1.jpeg]
